# Death, dying and informatics: misrepresenting religion on MedLine

**DOI:** 10.1186/1472-6939-6-6

**Published:** 2005-07-01

**Authors:** Pablo Rodríguez del Pozo, Joseph J Fins

**Affiliations:** 1 Division of Medical Ethics, Weill Medical College of Cornell University in Qatar (WCMC-Q). P.O. Box 24144, Education City, Doha, Qatar; 2 Division of Medical Ethics, Weill Medical College of Cornell University (WMCCU), New York, USA

## Abstract

**Background:**

The globalization of medical science carries for doctors worldwide a correlative duty to deepen their understanding of patients' cultural contexts and religious backgrounds, in order to satisfy each as a unique individual. To become better informed, practitioners may turn to MedLine, but it is unclear whether the information found there is an accurate representation of culture and religion. To test MedLine's representation of this field, we chose the topic of death and dying in the three major monotheistic religions.

**Methods:**

We searched MedLine using PubMed in order to retrieve and thematically analyze full-length scholarly journal papers or case reports dealing with religious traditions and end-of-life care. Our search consisted of a string of words that included the most common denominations of the three religions, the standard heading terms used by the National Reference Center for Bioethics Literature (NRCBL), and the Medical Subject Headings (MeSH) used by the National Library of Medicine. Eligible articles were limited to English-language papers with an abstract.

**Results:**

We found that while a bibliographic search in MedLine on this topic produced instant results and some valuable literature, the aggregate reflected a selection bias. American writers were over-represented given the global prevalence of these religious traditions. Denominationally affiliated authors predominated in representing the Christian traditions. The Islamic tradition was under-represented.

**Conclusion:**

MedLine's capability to identify the most current, reliable and accurate information about purely scientific topics should not be assumed to be the same case when considering the interface of religion, culture and end-of-life care.

## Background

With the globalization of medical science, there is a concomitant need to better understand cross-cultural differences [1]. This ethical imperative is perhaps most critical during life's final chapter when diverse populations invoke their religious traditions and doctrines to questions of death and dying [2,3]. To meet the needs of dying patients and their families, practitioners may turn to the convenience of databases like MedLine to provide more culturally-competent care. Although MedLine has become the gold standard for scientific papers, it is less clear that it is a comprehensive source for scholarship in the broader medical humanities [4]. In this paper we sought to determine how accurate MedLine is as a source of information about how religious beliefs inform end-of-life care[5,6].

To answer these questions we have queried MedLine to assess the scope and quality of information that those conducting a search might obtain using common search phrases that working practitioners might employ. Our objectives are twofold. First, to parse out MedLine's representation of reality from wider scholarly treatment concerned with the interface of religion, medicine and death and dying. Second, to stress that MedLine's reliability is related not only to what is indexed in it, but also to how it is accessed. Our topic is more cultural than scientific: death and dying in the three major monotheistic religions. This type of issue is particularly pertinent to healthcare providers treating a heterogeneous population, since culture and religion play a central role in shaping everything from patients' notions of health and disease causation, [7-10] to their reaction to pain, to their expectations of the doctor-patient relationship [11-14].

Compiling the literature on religion and medicine raises questions about the bibliographic strengths of informatic catalogues in general [15] and MedLine in particular when we seek less scientifically informed knowledge. It also challenges us to consider how user-dependent MedLine is when the search is less than straightforward and what the implications are of incomplete or decontextualized searches. Finally, it generates doubts about how theological issues are catalogued in a database designed primarily for scientific literature.

## Methods

We searched MedLine using PubMed in order to retrieve and thematically analyze full-length scholarly journal papers or case reports dealing with religious traditions and end-of-life care. Eligible papers were limited to English-language articles that included an abstract, to ascertain the articles' content prior retrieval. Editorials, book reviews, letters-to-the-editor and brief comments were excluded, because generally they are not peer-reviewed.

To assess the relative magnitude of the broader literature, a search without language or genre limitations was also run. The search was limited to articles published between January 1993 and June 30, 2004, with the actual search completed on July 2, 2004.

We defined our search as a string of words, connected by the appropriate syntax and Boolean operators [16]. The search would consist of two parts. The first contained the names of each of the three religious traditions, according to the Medical Subject Headings (MeSH) [17], the National Library of Medicine's thesaurus of terms used to index all articles in MedLine, as noted:

• christianity OR christian

• islam OR islamic OR muslims

• judaism OR jewish

The second part included the sub-strings "death and dying" and "terminal care". The former is the heading term used by the National Reference Center for Bioethics Literature (NRCBL) [18] as the higher-level descriptor for articles dealing with an ample range of issues related to death and end-of-life care. "Terminal care" is the higher-level term MeSH uses to classify those entries that deal with death and dying. PubMed automatically recognizes MeSH terms, and retrieves all articles classified under such terms. To summarize, then, the search strings were:

• (islam OR islamic OR muslims) AND ("death and dying" OR "terminal care")

• (christianity OR christian) AND ("death and dying" OR "terminal care")

• (judaism OR jewish) AND ("death and dying" OR "terminal care")

Eligible articles were assessed for publication characteristics including site of publication and country of origin as well as authorship and any institutional or denominational affiliation. Professional backgrounds of authors were also recorded from the papers' institutional websites or their home pages.

Articles that were the product of a logical – but nonsensical – Boolean hit, such as the retrieval of articles by authors with a surname of "Christian" or "Islam" or writers who worked at a "Jewish Hospital", were removed from consideration if they had nothing to do with death and dying. All other articles retrieved were studied in order to avoid the introduction of a selection bias.

The initial general searches for religion, death and dying and denomination are noted in Table 1. Narrowing the search as described above, we obtained the results condensed in Table 2.

**Table 1:** Articles retrieved by non-specified searches

String	English, with abstracts	All languages, with abstracts
Religion	6212	6894
"death and dying" OR "terminal care"	2704	3100
christianity OR christian	7492	8494
islam OR islamic OR muslims	1031	1160
judaism OR jewish	7144	7306

**Table 2:** Articles retrieved by the specific searches

String	English, with abstracts	All languages, with abstracts	%
(christianity OR christian) AND ("death and dying" OR "terminal care")	46	50	61.3
(islam OR islamic OR muslims) AND ("death and dying" OR "terminal care")	9	9	12
(judaism OR jewish) AND ("death and dying" OR "terminal care")	20	23	26.3
Total	75	82	99.6

## Results

### Main findings

The search retrieved a total of 75 references. After eliminating duplicative results (four) and nonsensical retrievals (five), 66 articles were available for analysis. These articles were published in 36 different journals from seven countries or regions, but U.S.-edited journals accounted for the majority, with 27 publications, followed by the United Kingdom with three. Other English-speaking and European countries were represented with one or two journals at most. There was one Israeli journal and none from the Arab League or other predominantly Muslim country.

The majority of articles were in non-denominational journals, although no major medical journal such as *The New England Journal of Medicine*, *The British Medical Journal*, *The Journal of the American Medical Association, The Lancet *or *Annals of Internal Medicine*, were represented in our sample. Only two were from denomination affiliated journals: *Health Progress*, which is the official organ of The Catholic Health Association of the United States, and *Christian Bioethics*, an interdenominational, non-ecumenical publication [19]. These two denominational journals concentrated almost a third of all articles retrieved (or 21 articles). Twelve articles reviewed Christian traditions, with a high concentration in the Catholic doctrine (seven articles). Eight articles dealt with Jewish teachings, while four discussed Islamic beliefs. No articles for the Islamic tradition were written by clergy. Nearly all of the articles (61 total) had identified authors. The remaining five did not have an identified author and formed part of a series of doctrinal articles published in *Health Progress*. More than a third of articles were authored by physicians, with another 16% written by nurses, thus indicating that health professionals accounted for almost two-thirds of retrieved articles. The remaining articles were written by authors working in ethics, the humanities, social work, theology or the law. Nine authors were identified as clergy. Eight were noted as Christian clerics (six Catholics and two Orthodox), including one nun. One author was noted to be a rabbi. There were no Islamic clerics identified amongst the writers.

Most authors (74%) were affiliated with hospitals or universities, and more than half (56%) were based in the U.S. The rest were evenly distributed among Europe, Canada and other English-speaking countries. Only one author was based in a Muslim country (Pakistan). Most authors (25 total) belonged to non-denominational institutions, while 17 were affiliated with Christian institutions, predominantly Catholic (11 total), evenly distributed among Catholic universities and hospitals. Another 12 authors belonged to Jewish institutions, eight from Jewish hospitals or hospices and another four from universities.

With this data we then tried to assess what practices would be prohibited, obligatory and permitted according to the three monotheistic traditions as represented in the retrieved literature. Nevertheless, our efforts led to identifying papers that failed to provide more than broad categoricals within the traditions. This obscures the rich debate that occurs in theological and scholarly circles over diverse questions such as pain management and the potential hastening of death; whether suffering is redemptive; artificial nutrition and hydration; medical futility; and the role of quality-of-life considerations, to name but a few contentious topics that involve a broader scholarly community than those whose work is cited on MedLine.

### Limitations

Our inquiry is limited by the search methods we employed as well as the MedLine database queried. However, our objective was, precisely, to identify such limitations in order to demonstrate the need for caution when excessively relying on database searches for topics which transcend the purely scientific.

Having noted this caveat, our study was limited by circumscribing eligible articles to those with abstracts, but this was necessary to identify articles that merited additional analysis. Our findings are only applicable to articles in English, the predominant language of MedLine, and cannot be generalized to other religious traditions or different thematic areas.

Finally, to avoid the introduction of our own biases about what would constitute an appropriate article for study, we included articles for analysis that would otherwise only marginally shed light on our designated areas of inquiry. This illustrates the limitations of relying purely on retrieved articles without an additional level of scholarly discrimination.

## Discussion

While MedLine is an invaluable source of information, our study indicates the potential limits of the scholarly convenience afforded by informatics. Our data suggest that users of MedLine will gain only a partial view of the range of scholarship related to death and dying and the three major monotheistic traditions. Although a bibliographic search in MedLine on death and dying in the teachings of the three major monotheistic traditions will produce instant results and some valuable literature, the price of such convenience may be a somewhat biased and partial view of the issue.

Although religion is present as a topic among the MedLine indexed literature, articles retrieved under "ethics" are three times more numerous than "religion". Despite the relatively large number of citations concerning religion, our study indicates that there was a general imbalance between the number of articles and followers of each of the traditions [20,21]. This discrepancy is even more obvious when MedLine is compared to the widely popular Google search engine. In contrast to the predominance of Christian and Jewish hits on MedLine, the same searches on Google showed more balanced numbers with respect to religion and death and dying: Christianity (38,400); Islam (11,200); and Judaism (19,500). The preponderance of Christian and Jewish articles on MedLine is multifactorial, and is a question that warrants additional study to understand the determinants which produce and disseminate scholarship in the art and science of medicine across differing religious and cultural traditions.

Whatever the tradition, the vast majority of articles came from journals based in the U.S. A few articles came from developed European countries, with one from Israel and another from South Africa. It is notable that, despite the presence of 400 medical journals in the WHO-designated Eastern Mediterranean region [22], which includes countries from Morocco to Pakistan, none of the articles we retrieved was from a journal based in a Muslim country. Authors' residences followed almost the same pattern.

This preponderance of U.S. journals and authors can alter the scholarly landscape and distort the application of religious teachings when articles are read outside of the North American context. Because these journals have global reach and influence, most articles they carry are firmly rooted in the American context, and cannot necessarily be extrapolated to the local environment. That may limit the literature's usefulness to those practicing in other parts of the world. And when practitioners respect uncritically these texts the way they do the scientific papers found in MedLine, this may lead to a misrepresentation of local cultures and traditions.

For example, in predominantly Catholic Latin America, the question of physician-assisted suicide or euthanasia – frequently addressed in these articles – may be little more than an intellectual curiosity, the practice of which is nearly inconceivable in a Latin context. No North American Catholic author wrote about death and dying in the context of poverty, which gives rise to the most difficult ethical quandaries from the Rio Grande to Antarctica [23]. Although questions about withholding and withdrawing advanced life-sustaining technologies is a frequent theme, it is a topic that is less than relevant when so many patients in the region do not have access to basic care. Questions about the sanctity of life in this context intersect questions of social justice and the relationship of poverty to coping with death, dying and suffering [24,25].

The fact that most articles were written by doctors and nurses may also contribute to a rather narrow focus on clinical practices at the end of life, ignoring broader public health and societal context factors that may influence the care provided to dying patients and their families. Papers featuring practical guidelines for the care of the dying Catholic [26,27] or Jewish patient [28] or the importance of family to the Muslim patient [29] can enhance doctors' cultural competence [30]. Nonetheless, these contextual pieces, so firmly situated in the clinic, do not do justice to broader questions about the healthcare system or the socio-economic organization. Nor do they explain the underlying theology. In our view, the latter limitation was not remediated by religious people writing many of the doctrinal articles. These articles were more theoretical than practical and often rigidly orthodox, unable to mollify religious scripture to accommodate the quotidian needs of patients and families at the end of life [31]. It is important to note that many of the leading pastoral care journals, which take this perspective, yielded no articles in our MedLine search, even though many are indexed.

## Conclusion

Although MedLine is an excellent source of the more *objective *reality of science, our observations indicate that MedLine is an incomplete source of information for the complex interplay of death, dying and religion.

These errors of omission reflect the orientation of the biomedical paradigm MedLine was meant to serve. Unfortunately, it has been unable to transcend its scientific origins.

## Competing interests

The author(s) declare that they have no competing interests.

## Authors' contributions

Both authors contributed equally to this work.

## Pre-publication history

The pre-publication history for this paper can be accessed here:


